# Direct and sensitive detection of a microsporidian parasite of bumblebees using loop-mediated isothermal amplification (LAMP)

**DOI:** 10.1038/s41598-020-57909-8

**Published:** 2020-01-24

**Authors:** Yuto Kato, Takahiro Yanagisawa, Madoka Nakai, Ken Komatsu, Maki N. Inoue

**Affiliations:** Department of Agriculture, Tokyo University of Agriculture and Technology, 3-5-8 Saiwai-cho, Fuchu Tokyo, 183-8509 Japan

**Keywords:** Infectious-disease diagnostics, Pathogens

## Abstract

The reduction of bumblebee populations has been reported in the last decades, and the microsporidian parasite *Nosema bombi* is considered as one of the factors contributing to such reduction. Although the decline of bee populations affects both wild plants and human food supply, the effects of *Nosema* spp. infections are not known because it is difficult to obtain infective spores from wild bees due to their low prevalence. Microscopical observation of fecal samples or midgut homogenates and/or PCR are generally used for *N. bombi* detection. However, the germination rate of microsporidian spore declines if they are kept at 4 °C for a long time or frozen. It is therefore crucial to minimize the diagnosis and isolation time of infective spores from field-collected samples. Therefore, we performed a loop-mediated isothermal amplification (LAMP) assay for the direct detection of *N. bombi* in bumblebee midgut homogenates. Using this method, we could detect *N. bombi* from individuals from which it was visible under the microscope and directly from wild individuals.

## Introduction

Pollinators play a crucial role in agricultural productivity and in the genetic diversity of wild plants^1,2^. Bumblebees are wild pollinators throughout temperate ecosystems, and their recent domestication has increased their economic importance^3^. Recently, the decrease of wild bumblebee populations has been reported worldwide^4–9^, and four species have become locally extinct in 11 European countries over the last 60 years^10^. Several factors have been proposed to contribute to this phenomenon, including the decline or fragmentation of their habitats, use of pesticides, and native and invasive pathogens. Within this scenario, the invasive microsporidian pathogen *Nosema bombi* has attracted attention^11^.

Microsporidia are obligatory intracellular parasites, belonging to kingdom fungi^12^. The host range of microsporidia species is broad as these can infect almost all vertebrate and invertebrate animals, with insects considered as one of the main host categories^13^. Microsporidia are generally opportunistic parasites, but they can cause severe symptoms to certain insect species^14,15^. *Nosema apis* and *N. ceranae* are considered parasites of the western honeybee *Apis mellifera*^16^ and eastern honeybee *A. cerana*^17^, respectively. Recently, *N. ceranae* was found to infect *A. mellifera* leading to the reduction of the survival rate of workers and collapse of colonies, suggesting that the host switch between *A. mellifera* and *A. cerana* contributed to the reduction of *A. mellifera* populations^18,19^.

Since the first report on *N. bombi* infecting bumblebees (*Bombus* spp.) in 1914^20^, several pathological studies have been conducted on this bumblebee/honeybee-parasite system, from parasite transmission^21^ and tissue tropism^22^, to the reduced fitness of hosts due to decreasing sperm production and reduced survival rate of workers^23^, and male longevity and colony size^24^. In the United States, *N. bombi* is considered as one of the factors causing the wild population decline of bumblebees because *N. bombi* prevalence was higher in the declining bumblebee species than in others^9,11^. However, little evidence has been reported on the negative effects of *N. bombi* infection on bumblebee species. To elucidate on this issue, a bioassay using a substantial amount of infectious *N. bombi* spores should be performed. However, it is difficult to isolate active spores from field samples. Firstly, the prevalence of *N. bombi* is often low in wild bumblebees. In the United States, the overall prevalence in nine different bumblebee species was 2.9% (*N* = 9909), and the highest prevalence was 37.2% (*N* = 172)^25^. In Spain, the mean prevalence was 1.2% (*N* = 83) in *Bombus terrestris*^26^, and in Japan, it was 7.5% (*N* = 120) and 0% (*N* = 100) in *B. terrestris* and native species, respectively^27^. Secondly, although the microsporidia prevalence can be high at the colony level, for example 61.8% of the commercial colonies were infected in Ireland^28^, wild nests of bumblebees are hardly found in the field because they are established under ground or beneath grasses. Finally, even if some active spores are isolated from wild bumblebees, the low stability of these spores often hinders further bioassays. Because the long-time storage and freezing of *N. apis* and *N. ceranae* spores reduce their infectivity^29^, this is also expected for *N. bombi* spores. Moreover, *N. bombi* is usually detected by microscopy of fecal samples or PCR assays, which are time-consuming methodologies, especially when many samples are examined.

Loop-mediated isothermal amplification (LAMP) is an isothermal gene amplification method of target DNA, which is inexpensive, easy, and rapid^30^. This method can be performed under isothermal conditions due to the strand displacement activity of *Bst* DNA polymerase, and it shows high specificity because the primer set used (F3, B3, FIP, and BIP) binds six regions of the target DNA. Two additional “loop-primers”, LF and LB, contribute to increase the specificity of the LAMP reaction and to accelerate it^31^. In this method, the amplification can be detected without electrophoresis using magnesium pyrophosphate as a by-product^32–34^. Another important feature of LAMP is that the *Bst* DNA polymerase is hardly affected by the most common inhibitors^35^, which enables amplification of a target sequence without DNA extraction^36,37^. In the present study, we performed LAMP for *N. bombi* detection and compared its sensitivity to that of conventional PCR. We also tested LAMP as a method for detecting *N. bombi* from midgut homogenates without DNA extraction, which improves the versatility of LAMP.

## Results

### Optimal LAMP reaction temperature

**Figure 1:**
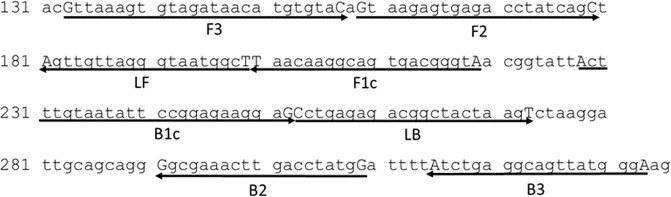
The LAMP primer set used in the present study. Right arrows indicate forward primers and left arrows indicate reverse primers. Terminals nucleotides are shown in capital letters. Numbers refer to nucleotide positions in the sequence of *Nosema bombi* SSU rRNA retrieved from GenBank (Accession No. AY008373).

**Figure 2:**
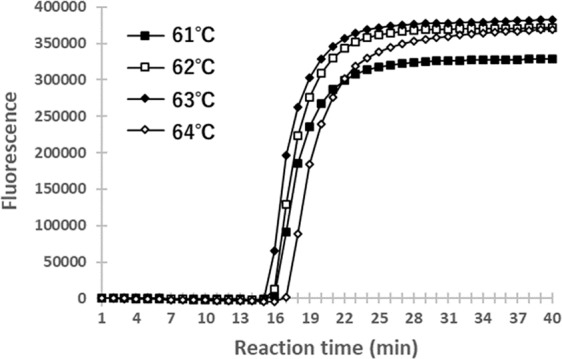
LAMP assay using different reaction temperatures. The reaction temperatures corresponding to each curve are indicated. This LAMP assay was performed for 40 min using 1 ng of DNA extracted from *N. bombi* spores. The vertical axis indicates the fluorescence signal and the horizontal axis the reaction time.

Our LAMP primer set was designed based on the SSU rRNA nucleotide sequence of *N. bombi* (GenBank Accession No. AY008373; Fig. 1). To optimize the reaction temperatures of the LAMP assay for detecting *N. bombi*, we conducted a LAMP assay from 61 °C to 64 °C using the DNA extracted from the spore suspension of *N. bombi* (c. 1 ng/μl). The fluorescence started to increase most rapidly at 63 °C, at about 15 min of reaction time (Fig. 2). Experiments were conducted three times with similar results. It should be noted that fluorescence started to increase within 20 min for all temperatures, indicating that the LAMP assay developed in this study had tolerance to slight temperature changes. In the following experiments, we performed LAMP at 63 °C.

### Sensitivity of PCR and LAMP assays

**Figure 3:**
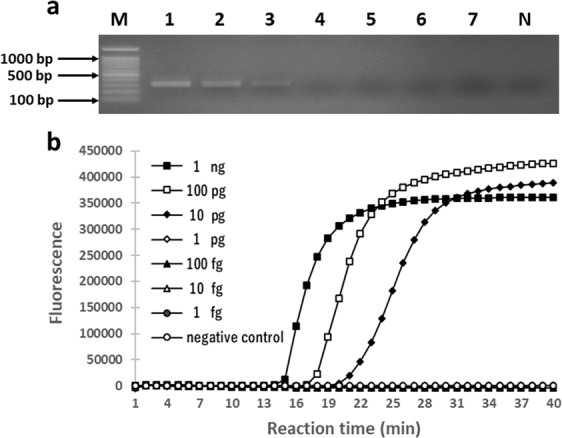
Sensitivity of the PCR and LAMP assays. (**a**) PCR assay; Numbers 1 to 7 indicate the samples resulting from the 10-fold serial dilutions with DNA concentrations ranging from 1 ng/μl to 1 fg/μl. M and N are the DNA marker and the negative control, respectively. (**b**) LAMP assay; the vertical axis indicates the fluorescence signal and the horizontal axis the reaction time. The LAMP was performed at 63 °C for 40 min. 1 μl each of DNA was used in both PCR and LAMP. Full-length gel is presented in Supplementary Fig. S1.

We compared the sensitivity of the LAMP assay to that of the PCR assay using 1 μl of each 10-fold serial dilution of the DNA template (1 ng/μl to 1 fg/μl) from *N. bombi* spore suspension and the primers for *N. bombi* SSU rRNA designed by Klee *et al*.^38^. Both PCR and LAMP assays could detect *N. bombi* using 10 pg of template DNA (Fig. 3). Therefore, the PCR assay using the *N. bombi*-specific primers and our LAMP assay showed the same sensitivity.

### Specificity test of LAMP primer

**Figure 4:**
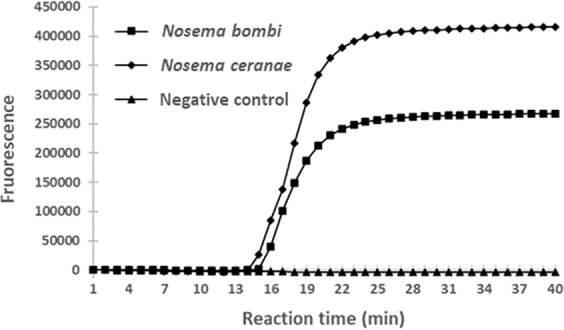
Specificity of our LAMP primer. The LAMP assay was performed at 63 °C for 40 min using *N. bombi* and *N. ceranae* DNA extracted from their spore suspensions. The DNA concentrations were 1.0 and 1.7 ng/μl, respectively. 1 μl of DNA was used in the LAMP assay. The vertical axis indicates the fluorescence signal and the horizontal axis indicates the reaction time.

In bumblebees, the infections of *N. ceranae* has been reported in Uruguay^39^ and South America^40^, and thus the specificity of our lamp primer set was examined using both *N. bombi* and *N. ceranae* DNA (the DNA concentrations were 1.0 and 1.7 ng/μl, respectively). Our LAMP primer set could detect not only *N. bombi* but also *N.ceranae* (Fig. 4).

### Detection of *N. bombi* by the direct LAMP assay and microscopy

**Figure 5:**
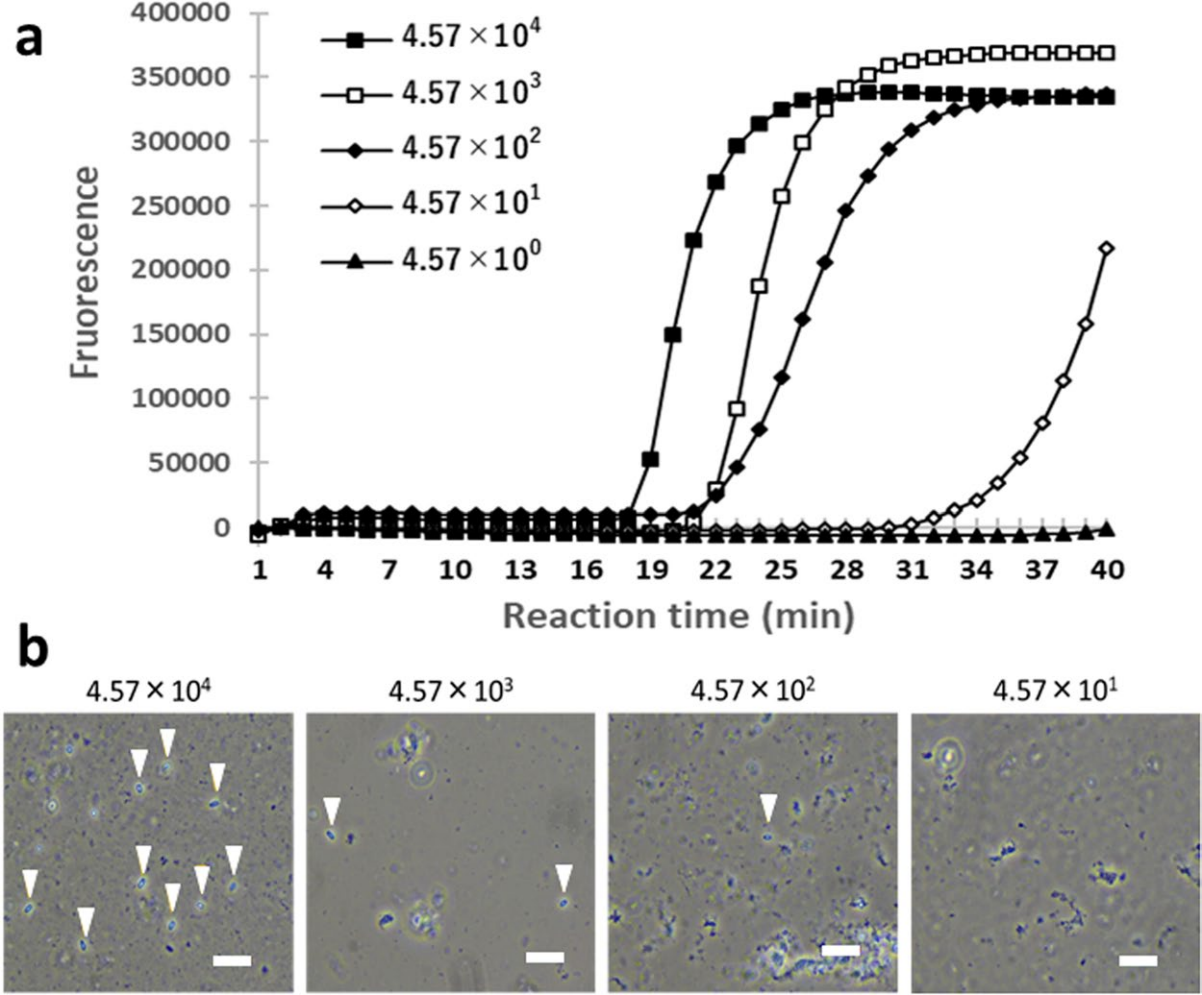
Detection limit of the direct LAMP assay using midgut homogenates as templates. (**a**) Direct LAMP assay using midgut homogenates. The vertical axis indicates the fluorescence signal and the horizontal axis the reaction time. Numbers indicate spore concentrations (spores/μl). LAMP was performed at 63 °C for 40 min. (**b**) Images of the artificially-infected midgut homogenates obtained by phase contrast microscopy (×400). Numbers indicate spore concentrations (spores/μl). Arrowheads indicate *N. bombi* spores. Scale bar = 20 μm. In direct LAMP assay, midgut homogenates were added using toothpicks, while in microscopic observation, 1 μl each of midgut homogenate was observed.

To apply LAMP for direct *N. bombi* detection without DNA extraction, we tested if LAMP could detect *N. bombi* from midgut homogenates and compared its result with that of microscopical observation. In this experiment, the artificially-infected midgut homogenates with spore concentrations ranging from 4.57 × 10^4^ spores/μl to 4.57 spores/μl were prepared, and then 1 μl of them were used for microscopic observations. In the direct LAMP assay, the samples were added to reaction reagent using toothpicks. This is because in the direct LAMP assay, no signal was observed when 1 μl of midgut homogenate was used (data not shown). The direct LAMP assay detected *N. bombi* from homogenates at concentration as low as 4.57 × 10^1^ spores/μl (Fig. 5a). On the other hand, using microscopy, *N. bombi* was detected from homogenates containing 4.57 × 10^2^ spores/μl, but not from homogenates with lower concentrations (Fig. 5b). Noteworthy, it took more than 5 min to find at least one spore even in the midgut homogenate with 4.57 × 10^2^ spores/μl by microscopy. This suggested that we were not able to find spores in midgut homogenates with approximately 100 spores/μl by microscopical observation, whereas LAMP could reliably detect *N. bombi* spores in homogenates with concentrations lower than this. Considering these results, direct LAMP can detect *N. bombi* in samples in which *N. bombi* spores could be observed under a microscope. On the contrary, in conventional PCR, amplifications did not occur even when homogenate with the highest spore concentration, which was added using a toothpick, was used, probably due to impurities.

### Direct detection of *N. bombi* from wild bumblebees

**Figure 6:**
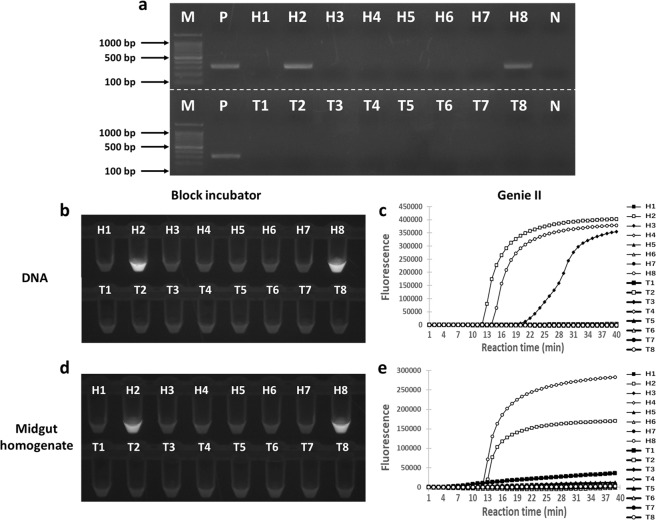
Detection of *N. bombi* by PCR and LAMP assays using wild individuals. (**a**) PCR assay using extracted DNA (from 5 to 10 ng/μl). (**b**) LAMP assay using extracted DNA and a block incubator. (**c**) LAMP assay using extracted DNA and the Genie^®^ІІ. (**d**) Direct LAMP assay using midgut homogenates and a block incubator. (**e**) Direct LAMP assay using midgut homogenates and the Genie^®^ІІ. Each LAMP assay was performed at 63 °C for 40 min. Sample names starting with “H” correspond to *B. hypocrita sapporoensis* samples and with “T” to *B. terrestris* sample; M: DNA marker, P: positive control, N: negative control. 1 μl each of DNA was used in (**a–c**), while the midgut homogenates were added using toothpicks in (**d,e**). Full-length gel is presented in Supplementary Fig. S2.

To confirm that direct LAMP could detect *N. bombi*-infected individuals collected in the field, we used the midgut homogenates of eight *B. hypocrita sapporoensis* and eight *B. terrestris* collected from the field for direct LAMP. To validate the results obtained by direct LAMP, we performed a PCR assay using 5 to 10 ng/μl each of template DNA extracted from each midgut sample, as well as normal LAMP also using template DNA. The PCR assay detected *N. bombi* from two out of the 16 individuals, H2 and H8 (Fig. 6a), and *N. bombi* infection was verified by direct sequencing analysis. *Nosema bombi* was also detected in these two individuals in the LAMP assay using template DNA by both Genie^®^II and block incubator (Fig. 6b,c). Moreover, similar results were obtained for midgut homogenates using a block incubator and Genie^®^II (Fig. 6d,e), indicating that LAMP can directly detect *N. bombi* from wild individuals. Under the microscope, *N. bombi* spores were observed in the midgut homogenates of H2 and H8 with spore concentrations of 6.25 × 10^5^ spores/μl and 3.00 × 10^5^ spores/μl, respectively. On the other hand, in H3 in Fig. 6c, no spores were found by microscopic observation; the PCR and the other LAMP results (Fig. 6a,b,d,e) were also negative. This LAMP assay using extracted DNAs was repeated once more; H3 was negative but T7 became positive, while H2 and H8 showed positive (Supplementary Fig. S3a). To further confirm the conclusion that only H2 and H8 were infected by *N. bombi*, we conducted an additional LAMP assay using these four samples (H2, H3, H8, and T7), two randomly selected samples (T1 and T5), a positive control (the same as for the PCR assays in this paper), and a negative control (ultra-pure water) (Supplementary Fig. S3b). In this assay, *N. bombi* was not detected from H3 and T7. Additionally, to examine whether these four samples, H2, H3, H8, and T7, were infected with *N. ceranae*, we conducted PCR for detecting *N. ceranae* using the primer targeting *N. ceranae* SSU rRNA gene (Table 1), which assured that no sample was infected (Supplementary Fig. S4). The results found in H3 and T7 were sometimes observed in LAMP assays^37^, which could be the unreproducible amplifications, and such false positive results occurred more frequently when a template homogenate was used without dilution.

**Table 1:** Primer sequences used in PCR and LAMP assays.

	Primer	Sequence (5′ to 3′)	Reference
PCR	Nbombi-SSU-Jf1	CCATGCATGTTTTTGAAGATTATTAT	Klee *et al*. (2006)
	Nbombi-SSU-Jr1	CATATATTTTTAAAATATGAAACAATAA	
	218MITOC-FOR	CGGCGACGATGTGATATGAAAATATTAA	Martín-Hernández *et al*. (2007)
	218MITOC-REV	CCCGGTCATTCTCAAACAAAAAACCG	
LAMP	F3	GTTAAAGTGTAGATAACATGTGTAC	This paper
	B3	TCCCATAACTGCCTCAGAT	
	FIP	TACCCGTCACTGCCTTGTTAGTAAGAGTGAGACCTATCAGC	
	BIP	ACTTTGTAATATTCCGGAGAAGGAGCCATAGGTCAAGTTTCGCC	
	LF	AGCCATTACCCTAACAACT	
	LB	CCTGAGAGACGGCTACTAAGT	

To confirm the result of our direct LAMP assay with the conventional PCR and the microscopic observation, we performed additional direct LAMP assays using another 73 samples collected from the field. Twelve out of 73 samples showed positive results in the direct LAMP assay and spores were observed under the microscopy from 3 out of these 12 samples, while the other 9 samples were negative in both the microscopy and PCR (Supplementary Table S1). According to these results, there were no samples which were positive under the microscopy but negative in the direct LAMP assay; that is, our direct LAMP assay could perfectly detect *N. bombi* infected samples from which some spores were visible under microscopy. We also confirmed that the nine samples that were negative in both microscopy and PCR were not infected by *N. ceranae* (Supplementary Fig. S4).

## Discussion

In this study, a direct LAMP assay for detecting *Nosema* parasites from midgut homogenates of bees was established for the first time. *Nosema* spp. are considered as possible agents of the current decline observed in bees worldwide. We could reliably detect *N. bombi* from infected individuals by suspending their midguts in PBS and performing LAMP using this suspension as the template. Using LAMP, *N. bombi*-infected bumblebees can be easily and rapidly (in less than 1 h) detected. Moreover, LAMP sensitivity is identical to that of conventional PCR and higher than that of microscopical observation. However, our LAMP primer set could also detect not only *N. bombi* but also *N. ceranae* (Fig. 4). Moreover, unreproducible amplification occurred sometimes. In the future, a specific primer set for *N. bombi* detection targeting other *N. bombi* gene should be prepared and solutions that could reduce unreproducible amplification should be identified.

A previous study^41^ also used LAMP for detecting *N. apis* and *N. ceranae* infecting honeybees, but the template material was the total DNA extracted from infected bees. Thus, its results cannot be directly applied to a practical field diagnosis. Microsporidian spores generally have a thick chitinous cell wall^42^, which protects them from severe environmental conditions, such as inadequate temperature and relative humidity^43–45^. For this reason, the extraction of the large amount of DNA from *Nosema* spp. spores involves physical crushing via glass beads or liquid nitrogen, which is time-consuming and requires special equipment^11,46–48^. On the other hand, the direct LAMP method established here could amplify the target DNA without these extraction steps.

We also showed that LAMP can be performed using a simple incubator, which allows us to examine many samples whereas in Genie^®^II we can only examine 16 samples simultaneously. Moreover, the reaction stability of the LAMP reaction at several temperatures and the stability of LAMP reagents at room temperature^49^ might allow to diagnose the *Nosema* spp. infections using non-specific equipment, as in a water bath at 60 °C. Using the LAMP method developed in this study, we will be able to obtain infective *N. bombi* spores by screening wild bumblebees, without DNA extraction in the field, and examine the influence of *Nosema* spp. infections on bumblebee populations. Furthermore, the reaction time of our LAMP method could be shortened to 30 min because when the midgut homogenate from which we could detect *N. bombi* spores by microscopy was used as a template, amplification occurred within 30 min. This direct detection method can also be applied for screening *N. apis* and *N. ceranae* in honeybees using the previously published LAMP primer sets^41^. The results obtained here suggest that direct LAMP can also be applied to other pollinating bee and *Nosema* species and thus contribute to examine the causes underlying the decline in wild bee populations.

It remains unclear how LAMP can detect *N. bombi* DNA directly from midgut homogenates. One possible reason is the existence of a small amount of “naked” *N. bombi* DNA (uncovered by cell membranes) leaking into the midgut of bees from spores that either died from succumbing to host immunity or were in mid-reproduction.

Although LAMP-based diagnoses have been applied to several human and plant diseases, to plant quarantine, and to food inspection^50,51^, and direct LAMP has been able to detect not only viruses or fungi of plants but also fungal virus^37^, such methods are not necessarily applied to pathogen detection in the field. Thus, to apply LAMP in on-site surveys, future studies on the development of simplified, direct diagnosis methods are needed.

## Materials and Methods

### Preparation of the *Nosema bombi* and* Nosema ceranae* spore suspension

*N. bombi* spores used for the experiments were isolated from four queens of *Bombus hypocrita hypocrita* collected in Yamanashi prefecture, Japan, in September 2016, and *N. ceranae* spores were isolated from *A. cerana* workers collected from a wild colony in Tokyo, Japan, in April 2016, after confirming *N. bombi* and *N. ceranae* infection as described below. Firstly, each midgut was homogenized in 100 μl of phosphate-buffered saline (PBS; 1370 mM NaCl, 27 mM KCl, 81 mM Na_2_HPO_4_ 12H_2_O, and 14.7 mM KH_2_PO_4_; pH 7.4) (hereafter referred to as midgut homogenate) and filtered through a lens paper into a 15-ml tube. The filtrate was centrifuged at 3000 × *g* for 10 min at 20 °C and the resulting supernatant was removed. The precipitate was washed by resuspension with 1 ml PBS and further centrifuged under the same conditions until the pellet became white. Finally, the pellet was resuspended in 100 μl PBS (hereafter referred to as the spore suspension) and *N. bombi* spore concentration calculated using a hemocytometer was 4.57 × 10^4^ spores/μl.

After DNA extraction and PCR using this spore suspension, direct sequencing analysis was conducted as described below. The obtained sequence was aligned using MEGA 6.0^52^ and compared with the *N. bombi* and *N. ceranae* SSU rRNA sequence deposited in GenBank (http://www.ncbi.nlm.nih.gov/genbank/) under accession number AY008373 and XR002966746, respectively. These extracted DNA were used as a positive control in all PCR assays.

### DNA extraction

A sample of midgut homogenate (50 μl) or spore suspension (25 μl) was mixed with 250 μl DNAzol, (Molecular Research Center, Cincinnati, OH, USA) and 250 mg glass beads (Sigma-Aldrich, St. Louis, MO, USA), and homogenized using Precellys 24 (Bertin, Montigny-le-Bretonneux, France) at 5500 rpm for 20 s. The homogenate was transferred to a new tube and 250 μl DNAzol and 1 μl Proteinase K (20 mg/ml) (Merck Bioscience, Darmstadt, Germany) were added and mixed by inversion (25 times). After incubation for 1 h at 50 °C, the mix was centrifuged at 15,400 × *g* for 10 min at 4 °C, and 300 μl of the resulting supernatant was transferred to a new tube. After adding 150 μl of 99% ethanol, and mixing by inversion (50 times), a new centrifugation was performed (20,400 × *g* for 10 min at 4 °C). Then discarding the resulting supernatant, the pellet was washed twice with 70% ethanol, dissolved with 20 μl of sterilized distilled water (SDW), and incubated at 60 °C for 15 min. The DNA concentration was then quantified using NanoVue Plus (GE, Boston, MA, USA).

### Preparation of artificially-infected midgut homogenates

Ten-fold serial dilutions of the spore suspension were prepared and 1 μl of each diluted solution was added to 9 μl of midgut homogenate of *Bombus ardens ardens* workers, which were confirmed to be free of *N. bombi* by PCR. The concentration of these solutions, designated “artificially-infected midgut homogenates” ranged from 4.57 × 10^4^ to 4.57 spores/μl.

### Amplification and sequencing

The PCR assay for detecting *N. bombi* and *N. ceranae* was performed using 1.0 μl of 10× *Ex* *Taq* Buffer (TaKaRa, Shiga, Japan), 0.8 μl of 2.5 mM each dNTP mixture (TaKaRa), 0.2 μl of 10 μM each forward and reverse primers for Small subunit (SSU) rRNA^38,53^ (Table 1), 0.05 μl of 5 U/μl *Ex* *Taq* HS (TaKaRa), 1 μl of template DNA, and 6.75 μl of SDW. The PCR profile for detection of *N. bombi* comprised initial denaturation at 94 °C for 3 min, followed by 40 cycles at 94 °C for 30 s, 50 °C for 30 s, and 72 °C for 30 s, and a final extension at 72 °C for 5 min. For detection of *N. ceranae*, the same PCR profile described by Martín-Hernández *et al*.^53^ was used. After electrophoresis and observation under UV light, the PCR products were purified using a QIAquick PCR Purification Kit (Qiagen, Hilden, Germany). The purified DNA was used for direct sequencing reactions with BigDye Terminator Kit ver. 3.1 (Applied Biosystems, Foster City, CA, USA). Sequencing was performed in the 3700 DNA analyzer (Applied Biosystems). The obtained sequences were confirmed as the *N. bombi* and *N. ceranae* SSU rRNA.

### LAMP

All LAMP experiments were conducted in a clean bench with autoclaved equipment and filter chips. The primer set used in the present study was designed using Primer Explorer 5 (https://primerexplorer.jp/) (Fig. 1 and Table 1). The LAMP reaction was performed using 12.5 μl of 2 × Premix [0.5 μl Tris-HCl (pH 8.8), 0.25 μl 1 M KCl, 0.25 μl 1 M (NH_4_)_2_SO_4_, 0.2 μl 1 M MgSO_4_, 0.25 μl 10% Tween20 (Wako, Osaka, Japan), 0.35 μl of each 100 mM dATP, 100 mM dTTP, 100 mM dCTP, and 100 mM dGTP (Promega), 4 μl of 5 M Betaine (Sigma-Aldrich), and 5.65 μl SDW], 5.0 μl of 5 × Primer mix (F3: B3: FIP: BIP: LF: LB: DW = 1: 1: 8: 8: 4: 4: 74; the concentration of each primer was 100 μM), 1.0 μl of Fluorescent Detection Reagent (Eiken-Chemical, Tokyo, Japan), 1.0 μl of 8 U/μl *Bst* DNA polymerase (NipponGene, Tokyo, Japan), 1.0 μl of template DNA, and 4.5 μl of SDW. When the midgut homogenate was used as the template, it was added to the LAMP reaction solution using a toothpick to reduce contamination. The reaction was performed using the Genie^®^ІІ (OptiGene, West Sussex, NJ, USA) at 63 °C for 40 min and terminated by heating at 98 °C and then cooling to 80 °C at 0.05 °C/s.

### Collection of wild individuals and infection diagnosis by PCR and LAMP

Workers of *B. terrestris* and *B. hypocrita hypocrita* (eight of each species) were collected at Biei-Cho, Hokkaido, Japan in August 2018. Direct LAMP was conducted using their midgut homogenates, which was added to the reaction reagent using a toothpick, and PCR and LAMP assays were conducted using 1 μl of extracted DNAs (c. 5–10 ng/μl) from them. In this experiment, LAMP was performed using both the Genie^®^II and a block incubator (BI-535, ASTEC, Fukuoka, Japan). When the block incubator was used, the fluorescence of LAMP products were checked under UV light.

The additional direct LAMP assays using 73 *B. hypocrita hypocrita* collected in Koshu City, Yamanashi, Japan, were conducted using their midgut homogenates added to the reaction reagent using a toothpick. Furthermore, PCR using 1 μl of extracted DNA and microscopy using 1 μl of midgut homogenates were also conducted. There were no replicates for this additional experiment.

## Supplementary information

Figure S1, Figure S2, Figure S3, Figure S4, Table S1.
