# Supplementary material for: Direct and sensitive detection of a microsporidian parasite of bumblebees using loop-mediated isothermal amplification (LAMP)
Source: Sci Rep. 2020 Jan 24;10:1118. doi: 10.1038/s41598-020-57909-8 (PMC6981208; doi:10.1038/s41598-020-57909-8)
Supplement: Supplementary file 1 — Figure S1, Figure S2, Figure S3, Figure S4, Table S1. [file 41598_2020_57909_MOESM1_ESM.pdf]

Title: Direct and sensitive detection of a microsporidian parasite of bumblebees using loop-mediated isothermal amplification (LAMP)

Yuto Kato, Takahiro Yanagisawa, Madoka Nakai, Ken Komatsu, Maki N. Inoue*

Department of Applied Biological Sciences, Tokyo University of Agriculture and Technology, 3-5-8 Saiwai-cho, Fuchu, Tokyo 183-8509, Japan

* Corresponding author: Maki N. Inoue, Department of Applied Biological Science, Tokyo University of Agriculture and Technology, 3-5-8 Saiwai, Fuchu, Tokyo, 183-8509, Japan

Tel.: +81 42-367-5619

E-mail: makimaki@cc.tuat.ac.jp

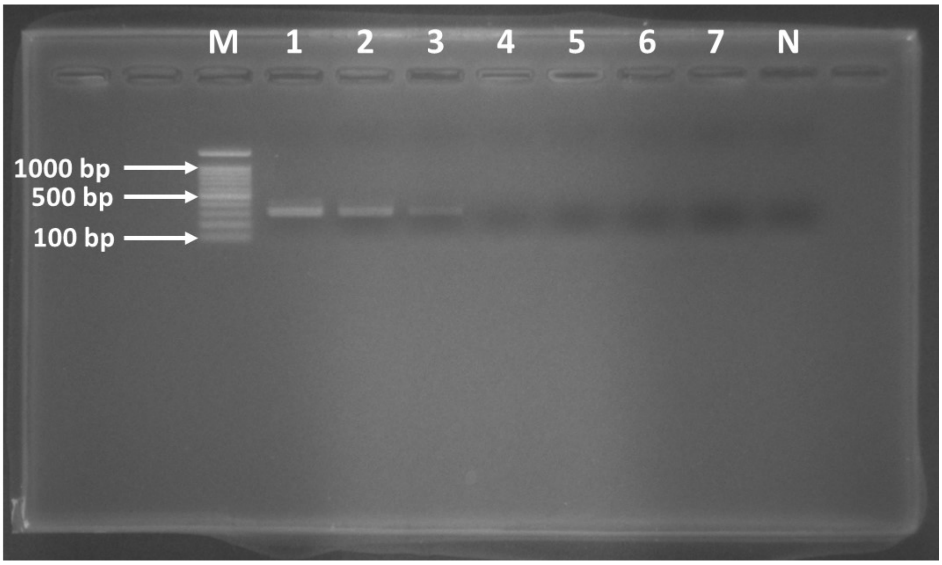

Figure S1. Full-length gel image of Figure 3a.

Numbers 1 to 7 indicate the samples resulting from the 10-fold serial dilutions with DNA concentrations ranging from 1 ng/µl to 1 fg/µl; M: DNA marker, N: negative control.

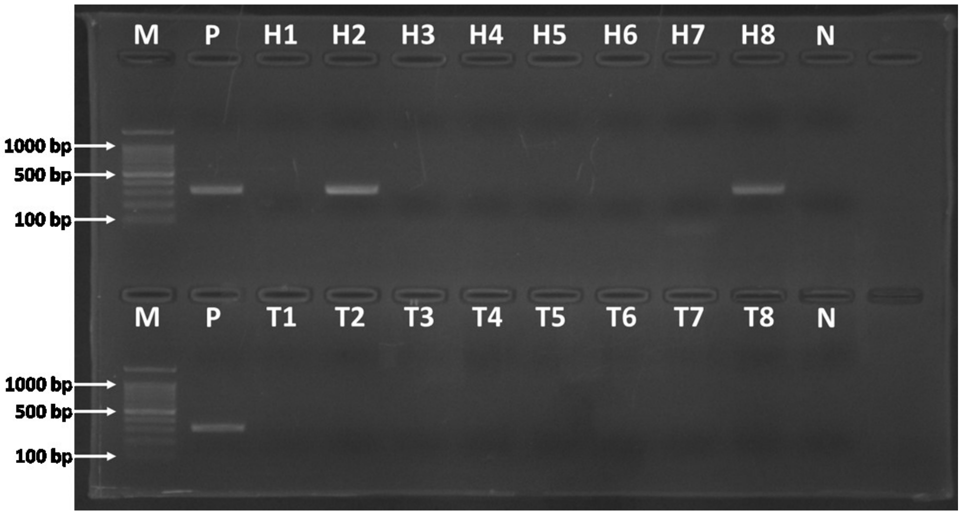

Figure S2. Full-length gel image of Figure 5a.

Sample names starting with “H” correspond to *B. hypocrita sapporoensis* samples and with “T” to *B. terrestris* samples; M: DNA marker, P: positive control, N: negative control.

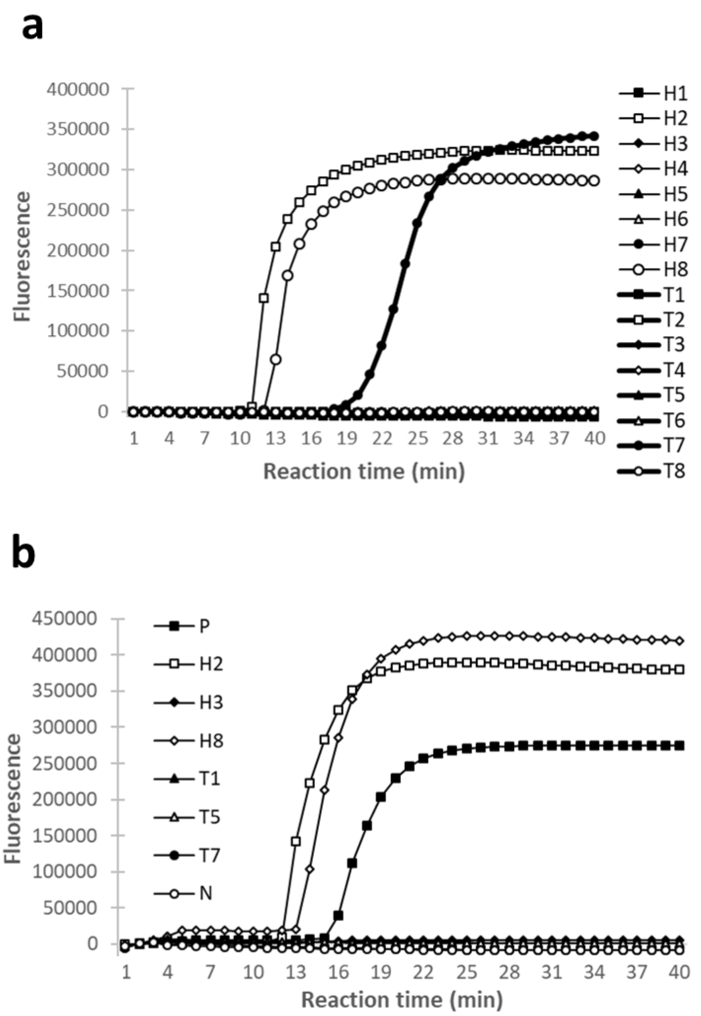

Figure S3. The result of the replicated and additional LAMP assay using extracted DNA

a. the replicated LAMP assay of Fig. 5c. b. the additional LAMP assay of Fig. 5c using positive samples, H2, H3, H8, and T7 and randomly selected samples, T1 and T5. LAMP was performed at 63 ºC for 40 min. P: positive control, N: negative control

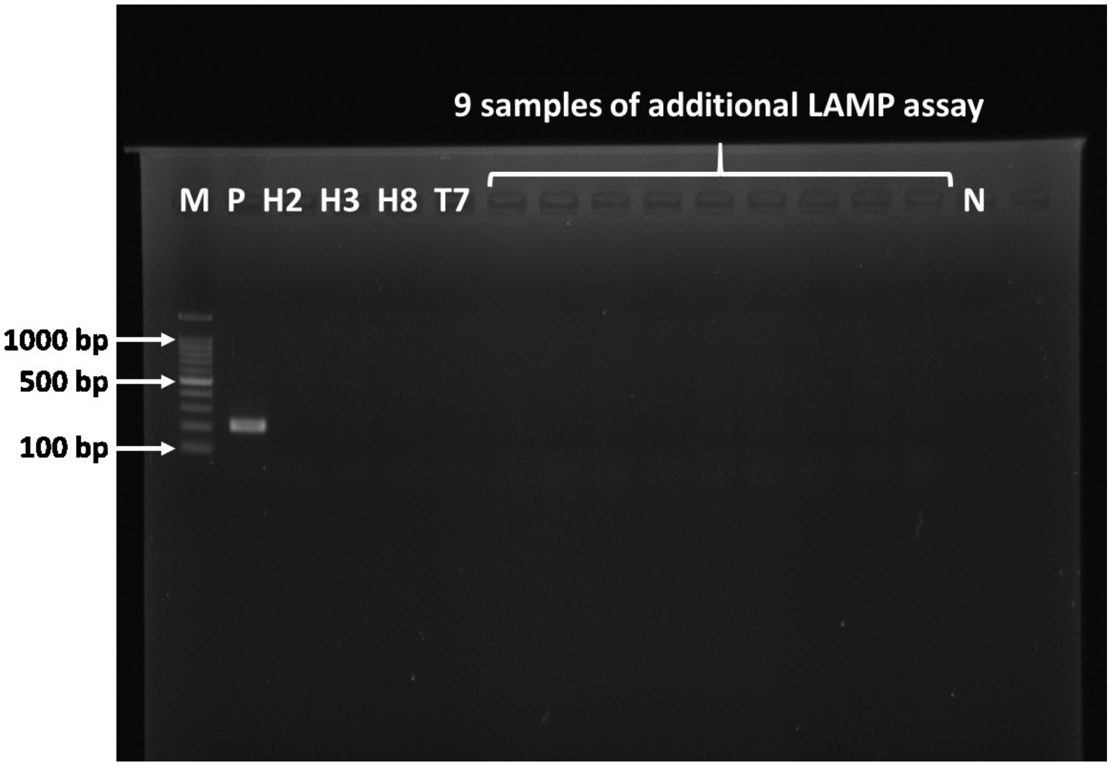

Figure S4. Screening of *Nosema ceranae* infections

The PCR gel image which confirmed *Nosema ceranae* infections using 14 samples. M: DNA marker, P: positive control, N: negative control.

Table S1. Detection of *Nosema bombi* from the additional field-collected samples

The direct LAMP and PCR assays, and the microscopic observation were conducted using 73 *Bombus hypocrita hypocrita* collected from Yamanashi, Japan. + indicates positive results and – indicates negative results.

	LAMP+			LAMP-		
N =73	total	microscopy+/PCR+	microscopy-/PCR-	total	microscopy+	PCR+
	12	3	0	61	0	0
